# Temporal Arteritis and Vision Loss in Microscopic Polyangiitis: A Case Report and Literature Review

**DOI:** 10.1155/2020/1426401

**Published:** 2020-03-26

**Authors:** Alexander G. Goglia, Michael Makar, Craig Vanuitert, Vadim Finkelstein

**Affiliations:** ^1^Rutgers Robert Wood Johnson Medical School, 675 Hoes Ln W, Piscataway, NJ 08854, USA; ^2^Rutgers Robert Wood Johnson Medical School, Department of Internal Medicine, 125 Paterson Street, New Brunswick, NJ 08901, USA; ^3^Penn Medicine Princeton Medical Center, Department of Internal Medicine, 1 Plainsboro Rd, Plainsboro Township, NJ 08536, USA; ^4^Penn Medicine Princeton Medical Center, Department of Pathology, 1 Plainsboro Rd, Plainsboro Township, NJ 08536, USA; ^5^Penn Medicine Princeton Medical Center, Princeton Hypertension and Nephrology, 1 Plainsboro Rd, Plainsboro Township, NJ 08536, USA

## Abstract

Microscopic polyangiitis (MPA) is an idiopathic autoimmune disease characterized by systemic vasculitis. While the lungs and kidneys are the major organs affected by MPA, it is known to involve multiple organ systems throughout the body. Temporal artery involvement is a very rare finding in MPA. This report presents a patient whose initial presentation was consistent with giant cell arteritis but was ultimately found to have microscopic polyangiitis. It highlights the importance of considering alternative types of vasculitis in the differential diagnosis for patients with atypical temporal artery biopsy findings.

## 1. Introduction

Primary vasculitides are a diverse group of disorders characterized by an autoimmune response against host blood vessels that often leads to multisystem disease [1]. These conditions have traditionally been classified into one of three categories based on the size of the vessels they affect: either large, medium, or small vessel vasculitides [2]. However, in rare cases, a primary vasculitis can involve vessels of various sizes, leading to heterogeneous and widely overlapping clinical features that require careful investigation.

Microscopic polyangiitis (MPA) is an idiopathic autoimmune disease characterized by systemic vasculitis [3]. It is a predominantly small vessel disease associated with antineutrophil cytoplasmic antibodies (ANCA), which are thought to play a causative role in its pathophysiology. While the lungs and kidneys are the major organs affected by MPA, it is known to involve multiple organ systems throughout the body. Notably, temporal artery involvement is a very rare manifestation of ANCA-associated vasculitides and is a finding more consistent with the large/medium vessel predominant vasculitis giant cell arteritis (GCA).

Here we report the case of a patient whose clinical picture was consistent with giant cell arteritis but was ultimately found to have microscopic polyangiitis. This case highlights the importance of identifying the proper diagnosis in these mixed vasculitides and provides the most up-to-date literature review in patients with temporal artery findings in MPA.

## 2. Case Description

An 81-year-old Caucasian male with hypertension and hyperlipidemia presented with one day of complete unilateral loss of vision in his right eye, along with jaw claudication, and bitemporal headaches. He reported 1 to 2 months of weight loss, worsening fatigue, decreased oral intake, and severe bilateral lower extremity pain and edema.

On physical exam, the patient was cachectic and ill-appearing with severe anasarca. He exhibited bilateral tenderness to gentle temporomandibular joint palpation, and visual exam revealed complete unilateral right-sided vision loss. Initial labs showed elevated ESR (64 mm/hr) and serum creatinine (11.9 mg/dL) levels, while urinalysis revealed moderate hematuria (10–20 RBCs/hpf) and proteinuria (urine protein/creatinine ratio of 1.15). Further testing revealed high-titer p-ANCA anti-myeloperoxidase IgG (79 AU/mL; normal, <19 AU/mL) antibodies and weakly positive antinuclear antibody titers (1 : 80). C-ANCA anti-proteinase-3 (PR3) and complement levels (c3, c4) were within normal limits.

**Figure 1:**
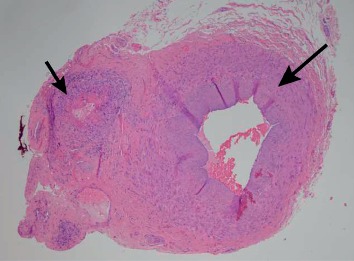
Temporal artery biopsy. The larger unaffected temporal artery can be seen on the right (large arrow) while the smaller arteriole involved by vasculitis (small arrow) extends to the left of the temporal artery.

**Figure 2:**
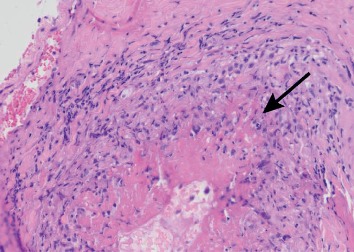
Vasculitis in arteriolar branch of the temporal artery. Extensive fibrinoid necrosis (arrow) and leukocytoclasis with vascular dilatation, destruction of the vascular wall, fibrin deposition, and inflammatory infiltrate in and around the vascular wall.

Given these findings, the patient was started on pulsed steroids and began to exhibit signs of clinical improvement. A subsequent right temporal artery biopsy (Figure 1) identified vasculitis with fibrinoid necrosis and leukocytoclasis in an arteriolar branch of the temporal artery (Figure 2). Furthermore, a chest CT scan identified multiple areas of ground-glass opacities involving the right upper and middle lobes and within the lingula, consistent with interstitial lung disease from a systemic vasculitis.

Based on these findings, the patient was diagnosed with microscopic polyangiitis with systemic involvement of the temporal and ophthalmic arteries. He was treated with three days of pulsed steroids and intermittent hemodialysis and was then transitioned to maintenance steroids. He received one dose of rituximab induction therapy in the hospital. Given his respiratory, cardiac, and hemodynamic instability, a renal biopsy was not obtained.

During his hospitalization, the patient developed acute hypoxic respiratory failure requiring BiPAP ventilation, likely secondary to his interstitial lung disease. In addition, he experienced repeated episodes of supraventricular tachyarrhythmia that required treatment with a chronotropic therapy (diltiazem drip). As his condition worsened, he was transferred to the ICU and ultimately developed an acute right-sided cerebral infarct with left hemiparesis. Given his multiorgan failure (respiratory failure and kidney failure) and stroke, his family chose to transition him to comfort care and he passed away in the hospital.

## 3. Discussion

The size of affected vessels has long been used to classify the various types of systemic vasculitis [2]. While these distinctions are informative for understanding the underlying pathophysiology, it is important to recognize that vessel involvement and thus clinical presentations can vary widely. Giant cell arteritis (GCA) is the most common systemic vasculitis in adults. Typical presentation includes involvement of temporal, vertebral, and ophthalmic arteries [4]. Involvement of the large arteries, such as the aorta, is less common. Clinical features are often vague, consisting of constitutional symptoms (e.g., fever, malaise, and weight loss) along with localized headache, temporal artery tenderness, and elevated ESR [4]. By comparison, microscopic polyangiitis (MPA) is a systemic small vessel vasculitis that often presents with renal and pulmonary manifestations and can, in rare cases, also involve medium and large caliber vessels such as the aorta [3].

Notably, it is exceedingly rare for MPA to present with temporal artery involvement, as only a small handful of such cases have been reported to date [5–13]. In our review of the literature, we identified only 14 cases of MPA reported to involve the temporal artery (Table 1). The true frequency of temporal artery involvement has been difficult to establish, but a recently published case series reported that ANCA-associated vasculitis occurred in <1% of patients with temporal artery biopsies (TAB) [5]. Cavazza et al. examined 871 patients with TAB and only identified three cases of MPA in 317 patients diagnosed with GCA. When considering a possible diagnosis of GCA, fibrinoid necrosis is a rare finding in the temporal artery and its presence should prompt the clinician to consider a broad differential diagnosis including MPA. While the patient's initial symptoms reflected temporal artery involvement, our findings demonstrate that it is important for clinicians to consider MPA as well as GCA. Given the inherent severity of MPA and other pauci-immune vasculitides, temporal artery biopsy findings may be used to help guide the treatment and management of patients with an unclear diagnosis. Further research studies are needed in this unique patient population.

## 4. Conclusion

Here, we describe a patient whose clinical presentation appeared to be entirely consistent with GCA (e.g., constitutional symptoms, bitemporal headaches with localized tenderness to palpation and jaw claudication, and elevated inflammatory markers), but whose biopsy results and further diagnostic workup revealed the presence of ANCA-positive MPA. This case is an important example of a difficult clinical presentation of small vessel vasculitis with temporal artery involvement in an older adult with unilateral vision loss and acute renal failure. It highlights the importance of considering alternative types of vasculitis in the differential diagnosis for patients with atypical temporal artery biopsy findings.

## Figures and Tables

**Table 1:** Literature review of temporal artery involvement in patients with MPA.

	Patients	Presenting symptoms	Organ systems involved	Biopsy findings
Cavazza et al. [5]	3/322	NA	NA	TAB: small-vessel vasculitis; no fibrinoid necrosis or leukocytoclasis

Chirinos et al. [6]	1	Constitutional symptoms (e.g., weight loss, weakness, fever, night sweats, myalgias), recurrent pulmonary infiltrates, no temporal arteritis	Renal, pulmonary, cutaneous, aorta	Palmar lesion: leukocytoclastic vasculitis
				Renal: pauci-immune necrotizing crescentic glomerulonephritis

Genereau et al. [7]	3/27	Headache (temporal), vision loss, jaw claudication, constitutional symptoms	Pulmonary, renal, URT, cutaneous, cardiovascular, PNS	TAB: fibrinoid necrosis in 3/3

Hamidou et al. [9]	3/120	Headache, jaw claudication, mononeuritis, scleritis, constitutional symptoms	Renal, middle ear, ocular, URT	TAB: fibrinoid necrosis in 2/3

Morinaga et al. [10]	1	Headache (temporal), dysphagia, hoarseness, dysgeusia, gait instability, cranial neuropathies	Renal, CNA	Renal: necrotizing glomerulonephritis with fibrinoid necrosis
				TAB: temporal arteritis, no giant cells

Planté-Bordeneuve et al. [11]	1	Upper extremity hemiparesis, jaw claudication, bitemporal headache with tenderness	Pulmonary, renal, muscular	TAB: transmural inflammation, focal zones of fibrinoid necrosis

Tanaka et al. [12]	1	Bitemporal pain/tenderness, fever	Renal, pulmonary (on relapse)	TAB: fibrinoid necrosis and luminal stenosis in surrounding SV
				Renal: pauci-immune necrotizing crescentic glomerulonephritis

Zuckerman et al. [13]	1^*∗*^	Headache, bitemporal tenderness, jaw claudication, visual disturbances	Renal (on follow-up presentation)	TAB: chronic inflammation. Within arterial wall, giant cells present
				Renal: pauci-immune necrotizing crescentic glomerulonephritis

TAB, temporal artery biopsy; URT, upper respiratory tract; PNS, peripheral nervous system; CNS, central nervous system; SV, small vessels. ^*∗*^GCA diagnosed, followed by diagnosis of renal-limited MPA 4 years later.
